# Superior esterolytic activity in environmental *Lactococcus lactis* strains is linked to the presence of the SGNH hydrolase family of esterases

**DOI:** 10.3168/jdsc.2020-0003

**Published:** 2020-10-29

**Authors:** Desirée Román Naranjo, Michael Callanan, Anne Thierry, Olivia McAuliffe

**Affiliations:** 1 Teagasc Food Research Centre, Moorepark, Fermoy, Cork, Ireland; 2 Cork Institute of Technology, Cork, Ireland; 3 VistaMilk SFI Research Centre, Moorepark, Fermoy, Cork, Ireland; 4 STLO, INRAE, Institut Agro, Rennes, France

## Abstract

*Lactococcus lactis* strains are widely used in the dairy industry in fermentation processes for production of cheese and fermented milks. However, the esterolytic activity of *L. lactis* is not generally considered high. For this reason, purified microbial lipases and esterases are often added in certain dairy processes to generate specific flavors in the final food product. This work demonstrates the superior esterolytic activity of a collection of *L. lactis* strains isolated from different environmental sources compared with that of dairy-derived strains. It provides further evidence of the more diverse metabolic capabilities displayed by *L. lactis* strains from environmental sources compared to their domesticated dairy counterparts. Furthermore, the presence of a 1,287-bp gene encoding a 428-amino acid SGNH hydrolase in the high-esterolytic environmental strains suggests a possible link between superior esterolytic activity and the presence of the esterase from the SGNH hydrolase family.

## Highlights

• *Lactococcus lactis* from environmental niches show high esterolytic activity
• Higher metabolic diversity is seen in environmental versus dairy L. lactis strains
• SGNH hydrolase family of esterases may be linked to high esterolytic activity

## Graphical Abstract

**Figure:**
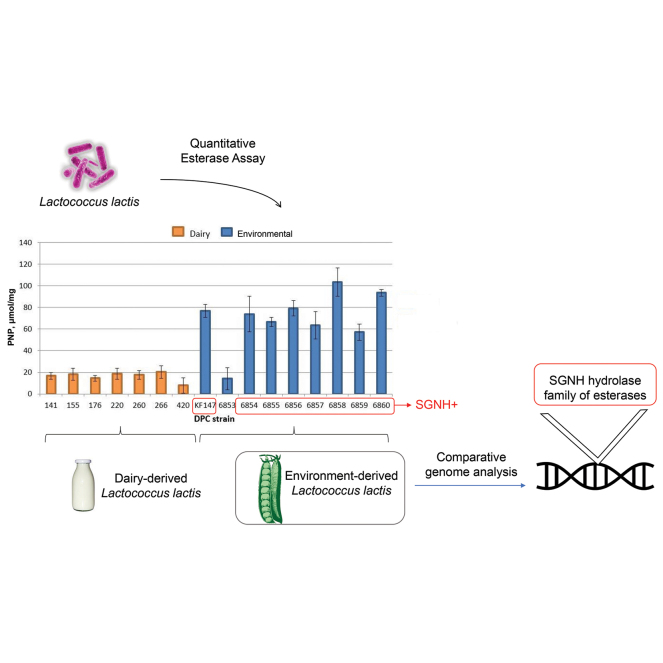
Summary: We investigated the esterolytic activity of dairy- and environment-derived *Lactococcus lactis* strains through a quantitative esterase assay based on hydrolysis of *p*-nitrophenyl dodecanoate (PNP). In general, environmental *L. lactis* strains had higher esterolytic activity than dairy strains. Comparative genome analysis revealed the presence of an open reading frame related to esterolytic activity in the environmental strain *L. lactis* DPC6855 (from corn), encoding the predicted product SGNH/GDSL hydrolase family protein. The 1,287-bp gene encodes a 428-amino acid SGNH/GDSL hydrolase. The presence of this gene in most of the environment-derived strains was established by PCR; the gene was not found in the genome of *L. lactis* DPC6853 or in genomes of *L. lactis* strains from dairy sources, suggesting a possible correlation between the SGNH hydrolase family and higher esterolytic activity. This work provides further evidence of more diverse genotypic and phenotypic traits in environmental compared with dairy *L. lactis* strains.

Lipolysis is an important biochemical event for flavor diversification in dairy manufacture. The pathway generates free fatty acids, di- and monoglycerides, and glycerol, which contribute to the flavor profile of fermented dairy products and act as substrates for other highly flavored components (Thierry et al., 2017). Many mold-ripened cheeses, such as blue cheese, undergo significant lipolytic activity through the actions of *Penicillium roqueforti*, whereby volatile and nonvolatile aroma compounds (mainly methyl ketones) are generated to provide unique flavors (Collins et al., 2003; Martín and Coton, 2017). The key enzymes involved in this lipolytic process are lipases and esterases, which catalyze hydrolysis and synthesis of esters and triglycerides, contributing to flavor development (Broadbent et al., 2005). The free fatty acids released by these enzymes act as precursors for flavor compounds such as esters, methyl ketones, lactones, and secondary alcohols (Thierry et al., 2017; McAuliffe et al., 2019).

Lactic acid bacteria, including *Lactococcus lactis*, are usually considered to have weak esterolytic activity compared with other bacterial species such as *Flavobacterium, Acinetobacter, Propionibacterium*, and *Pseudomonas* (Collins et al., 2003; Thierry et al., 2017). However, *L. lactis* strains isolated from environmental (or nondairy) niches exhibit much greater diversity in their metabolic capabilities than their dairy counterparts (Alemayehu et al., 2014; Cavanagh et al., 2014). Environmental lactococcal strains exhibit certain adaptation capabilities such as higher tolerance to salt and alkaline conditions, high glutamate dehydrogenase (GDH) activity, and diverse metabolization of carbohydrates, including sugars usually found in plant environments such as arabinose and xylose, which has been demonstrated to affect the production of flavor compounds in certain dairy processes (Alemayehu et al., 2014; Cavanagh et al., 2014, 2015). Although no significant difference was found in lipase production by dairy and nondairy *L. lactis* in a previous study (Nomura et al., 2006), Kalbaza et al. (2018) demonstrated higher lipolytic activity in nondairy *L. lactis* than in *Lactobacillus* strains.

In this study, we investigated the esterolytic activity of a group of 16 dairy and environmental *L. lactis* strains (Table 1). The dairy isolates were of the subspecies *lactis*, whereas all nondairy isolates, with the exception of DPC6853, were of the subspecies *cremoris*. However, we have shown in previous studies that environmental *L. lactis* strains that are genotypically subspecies *cremoris* behave phenotypically like dairy subspecies *lactis* (Cavanagh et al., 2015); therefore, we phenotypically compared the dairy subspecies *lactis* strains to the environmental subspecies *cremoris* strains. Cell extracts were prepared according to a method previously described (Stefanovic et al., 2017) from overnight cultures grown in M17 (Oxoid, Basingstoke, UK) supplemented with 5 g/L lactose monohydrate (L-M17; VWR, Leuven, Belgium) for dairy strains or M17 supplemented with 5 g/L d(+)-glucose monohydrate (G-M17; VWR) for environmental strains. A quantitative esterase assay, relying on the principle of hydrolysis of *p*-nitrophenyl dodecanoate (Sigma-Aldrich, Arklow, Ireland) to dodecanoic acid and *p*-nitrophenyl (**PNP**) was used (Bertuzzi, 2017). Although both dairy and environmental strains showed esterase activity, there were clear differences between the 2 groups of strains in relation to the levels of esterase activity. The dairy strains showed activities in the range of 10 to 22 μmol of PNP/mg, with a mean of 18.5 μmol PNP/mg (Figure 1). However, environmental strains showed the greatest activity (range of 58–100 μmol of PNP/mg; mean of 78.5 μmol of PNP/mg), except for strain DPC6853, the activity of which was 17.5 μmol of PNP/mg. The environmental strain used as a reference in our study, KF147, shared high esterase activity with the environment-derived group of strains. The means of the 2 groups analyzed (dairy and environmental) differed significantly (*P* = 0.00003). Our findings further confirm the more variable metabolic activities of environmental *L. lactis* strains (Alemayehu et al., 2014; Cavanagh et al., 2014, 2015). Interestingly, one environmental strain, DPC6853, did not show this high esterolytic activity. This strain was the only one in our collection isolated from corn, and further investigation is required to determine whether there is a link between the observed activity and specific environmental conditions.

**Table 1:** *Lactococcus lactis* strains used in this study

DPC^1^ code	Species/subspecies (ssp.)	Isolation source	Source or reference^1^	Accession no.
Dairy				
141	*L. lactis* ssp. *lactis*	Mixed-strain starter culture	DPC CC	
155	*L. lactis* ssp. *lactis*	Mixed-strain starter culture	DPC CC	
176	*L. lactis* ssp. *lactis* biovar *diacetylactis*	Mixed-strain starter culture	DPC CC	
220	*L. lactis* ssp. *lactis* biovar *diacetylactis*	Mixed-strain starter culture	DPC CC	
260	*L. lactis* ssp. *lactis*	Mixed-strain starter culture	DPC CC	
266	*L. lactis* ssp. *lactis*	Mixed-strain starter culture	DPC CC	
420	*L. lactis* ssp. *lactis*	Mixed-strain starter culture	DPC CC	
Nondairy				
6853	*L. lactis* ssp. *lactis*	Corn	Cavanagh et al. (2015); DPC CC	LAVD00000000.1
6854	*L. lactis* ssp. *cremoris*	Grass	DPC CC	
6855	*L. lactis* ssp. *cremoris*	Grass	Roman Naranjo et al. (2019); DPC CC	VERW00000000.1
6856	*L. lactis* ssp. *cremoris*	Bovine rumen	Cavanagh et al. (2015); DPC CC	LAVW00000000.1
6857	*L. lactis* ssp. *cremoris*	Grass	DPC CC	
6858	*L. lactis* ssp. *cremoris*	Grass	DPC CC	
6859	*L. lactis* ssp. *cremoris*	Grass	DPC CC	
6860	*L. lactis* ssp. *cremoris*	Grass	Cavanagh et al. (2015); DPC CC	LAVX00000000.1
KF147	*L. lactis* ssp. *cremoris*	Mung bean sprouts	Siezen et al. (2010)	CP001834

1 DPC CC = Teagasc DPC Culture Collection housed at the Teagasc Food Research Centre, Moorepark, Fermoy, Cork, Ireland.

**Figure 1:**
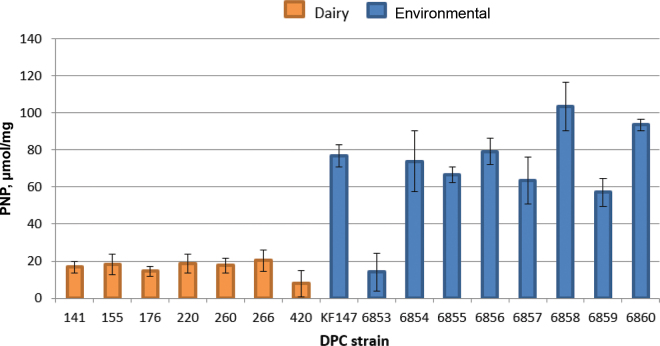
Esterase activity [expressed in μmol of *p*-nitrophenyl (PNP)/mg of cell-free extracts] of dairy and environmental *Lactococcus lactis* strains on the substrate *p*-nitrophenyl dodecanoate. The concentration of *p*-nitrophenyl released was determined from a standard curve obtained for a set of standards of *p*-nitrophenyl phosphate (0–500 nmol; Sigma-Aldrich, St. Louis, MO). The concentration of protein in each sample was calculated using the Qubit Protein assay kit (ThermoFisher Scientific, Waltham, MA). Experimental results from the different groups (dairy and environmental) were statistically examined by running a *t*-test (2 samples assuming equal variances) in Excel (Microsoft Corp., Redmond, WA) with an α value of 0.05. Error bars represent SD of 3 independent experiments. See Table 1 for sources of strains.

To determine a possible genetic link to the high esterolytic activity observed in the environmental strains, comparative genome analysis was performed on available genome sequences of the environmental strain set (Table 1). The genome data were analyzed using the Artemis 16.0.0 genome browser (Carver et al., 2005) and the BLASTP web server (Madden et al., 1996), using default parameters. Analysis of the draft genome of strain DPC6855, isolated from grass, revealed the presence of one open reading frame (FIB60_02895) related to esterolytic activity, which encodes the predicted product SGNH/GDSL hydrolase family protein (Figure 2A). Subsequent analysis of the genomes available for 3 other environmental strains in our collection also revealed the presence of this 1,287-bp gene encoding the 428-AA SGNH/GDSL hydrolase in DPC6856 and DPC6860. Examination of the literature revealed that the SGNH-hydrolase family is a recently classified subgroup of the GDSL group of esterase and lipase enzymes that possess multifunctional properties such as regiospecificity and broad substrate specificity (Akoh et al., 2004). A conserved XynE-like domain is associated with the SGNH hydrolase subfamily and has the consensus AA sequence of Ser-Gly-Asn-His (SGNH) found in the active site. This motif provides a catalytic mechanism different from the classical GxSxG motif-containing hydrolases, such as the lack of nucleophile elbow and the presence of a flexible active site (Akoh et al., 2004; Reina et al., 2007; Oh et al., 2019). The SGNH hydrolase encoded by FIB60_02895 is related to the putative arylesterase/acylhydrolase encoded by the *xynE* gene located in a xylanase gene cluster in the rumen microbe *Prevotella bryantii* (Miyazaki et al., 2003).

**Figure 2:**
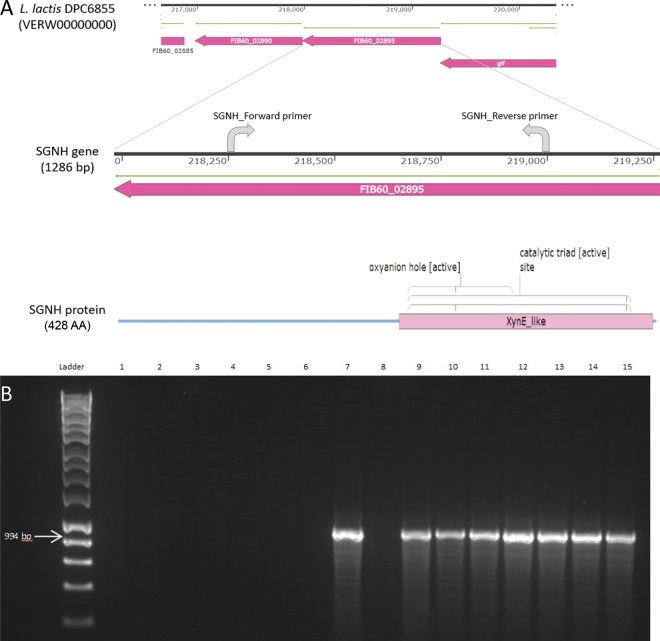
(A) Graphical representation of FIB60_02895 gene locus encoding SGNH hydrolase protein and surrounding genes [FIB60_02890 (glycosyl hydrolase) and *glf* (UDP-galactopyranose mutase)] from the draft genome sequence of *Lactococcus lactis* DPC6855 isolated from grass. Also shown are the locations of the SGNH_Forward and SGNH_Reverse primers used to generate the 994-bp product in the PCR-based assay. The predicted SGNH hydrolase protein is shown, revealing the location of the conserved domain “Xyn_E-like” as well as the catalytic triad site and oxyanion hole found within the enzyme. Graphic was generated using SnapGene software (Insightful Science; snapgene.com). (B) PCR-based detection of the SGNH gene in the dairy and environmental strain sets. A 1% agarose gel was used and HyperLadder 1 kb (Bioline, London, UK) was used as the molecular weight marker. The dairy strains are represented in lanes 1 to 6: DPC141 (1), DPC155 (2), DPC176 (3), DPC220 (4), DPC266 (5), DPC420 (6), and the environmental strains are represented in lanes 7 to 15: KF147 (7), DPC6853 (8), DPC6854 (9), DPC6855 (10), DPC6856 (11), DPC6857 (12), DPC6858 (13), DPC6859 (14), DPC6860 (15). See Table 1 for sources of strains.

Interestingly, this gene was not found in any of the publicly available genomes of *L. lactis* strains from dairy sources, or indeed, the genome of strain DPC6853 from corn, which displayed lower levels of esterase activity than the other environmental strains. The presence of the gene was confirmed in other environmental strains for which genome sequence information is publicly available, such as *L. lactis* ssp. *lactis* NCDO 2118 (isolated from frozen peas; Oliveira et al., 2014) and *L. lactis* ssp. *cremoris* KW2 (isolated from fermented corn; Kelly et al., 2013). Indeed, 2 loci encoding SGNH/GDSL hydrolase proteins were identified in strain KF147. The first is LLKF_RS02565, which encodes a GDSL family lipase. This 858-bp gene encodes a 286-AA protein and contains an Ypmr_like conserved domain. The second is a 1,286-bp gene (LLKF_0950) that encodes a 428-AA predicted protein product with 98% similarity to that found in DPC6855, DPC6856, and DPC6860 and described as a SGNH hydrolase superfamily protein, also related to esterolytic activity.

To determine the presence of the gene encoding SGNH hydrolase in the strains used in this study where whole-genome sequences are not yet available, we designed a set of primers, SGNH-F (5′-TGAGTGGTACGGCCTTTCGC-3′) and SGNH-R (5′-GAAAATAATCAATCAAGCACATACAT-3′), to amplify the partial gene sequence. No amplification product was detected in any of the dairy strains tested, whereas the 994-bp product was detected in all environmental strains except for the corn-derived strain with low esterase activity (DPC6853; Figure 2B). Subsequent sequencing of the amplified products revealed 100% identity to the gene found in DPC6855. Thus, except for the corn-derived DPC6853, all 7 environmental isolates in this study possessed the SGNH hydrolase gene, whereas it was not detected in any of the tested dairy strains using these PCR conditions. This correlates with our phenotypic analysis because the dairy strains showed low esterase activity compared with the environmental strains (<22 vs. ∼80 μmol of PNP/mg, respectively), confirming a link between the presence of the FIB60_02895 gene and higher esterase activity.

In conclusion, this work provides further evidence of more diverse genotypic and phenotypic traits in *L. lactis* strains from environmental sources compared with their dairy counterparts. An SGNH hydrolase protein was identified that is potentially related to the higher esterase activity observed in these strains, and work is currently ongoing to associate the role of this gene with the functionality observed. Alternative knockout methods are being tested because the traditional knockout by double recombination has proven ineffective in this case. In addition, the metabolite profiles and the ability of these environmental strains to hydrolyze milk glycerides compared with the less-active dairy strains are being examined. These strains represent potential options for in situ production of lipolytic enzymes in dairy processing.
